# Hepatitis C drug prescriptions and Medicaid policies--four states, Indian health care system, USA 2018

**DOI:** 10.1186/s12939-019-1101-4

**Published:** 2019-12-04

**Authors:** Brigg Reilley, Matt Miller, Matt Hudson, Rick Haverkate, Jessica Leston

**Affiliations:** 1 Northwest Portland Area Indian Health Board, 2121 Broadway Suite 300, Portland, OR 97201 USA; 2 United States Indian Health Service, 5600 Fishers Lane, Rockville, MD 20857 USA; 3 Johns Hopkins University School of Medicine, 733 N Broadway, Baltimore, MD 21205 USA

## Abstract

Medicaid, the state-level public insurance in the United States, has widely differing criteria treatment for hepatitis C virus (HCV) such as stage of liver fibrosis, documented sobriety, and specialist consultation. In a rural health network, facilities located in two less restrictive states prescribed HCV drugs at a significantly higher rate than two more restrictive states (rate ratio 4.7, CI 2.6–8.5). Prescription rates per population were highly associated with HCV treatment policies.

## Introduction

In the United States, an estimated 2.4 million persons have chronic hepatitis C virus (HCV) infection [1], and the number of deaths from HCV-related mortality is greater than those from HIV and TB combined [2]. Treatment of HCV with direct acting antivirals (DAAs) can cure over 95% of patients with HCV, and cure has been shown to greatly reduce liver-related as well as all-cause mortality [3, 4]. The medical benefits of early treatment of treatment of HCV, before any liver fibrosis has occurred, further improves medical outcomes for HCV patients [5]. However, access to treatment has been limited, often due to treatment criteria that are at least partially attributable to the cost of drugs [6].

American Indian and Alaska Native (AI/AN) people have over twice the national rate of HCV related mortality, making access to treatment among this population a priority [7]. Surveillance data from the Indian Health Service (IHS), the federal agency that provides direct medical care to AI/AN communities, documented approximately 30,000 unique patients with HCV, with significant differences in HCV burden by region [8].

The overall Indian health care system is comprised of federal (IHS), tribal, and urban Indian facilities. It is the largest health system provider to AI/AN communities, serving approximately 2.6 million persons in 37 states. State Medicaid programs are a key public insurer for an important proportion of patients in this health network. However, state Medicaid programs vary greatly in HCV treatment eligibility criteria, with some states requiring late stage liver fibrosis, specialist consultation, documented periods of sobriety and other qualifications prior to HCV treatment approval [9]. These eligibility requirements for treatment of a confirmed diagnosis of an infectious disease are thought to be unique to HCV, and are not in alignment with clinical recommendations [10].

We examined prescription data to determine if state Medicaid policy correlated with significant differences for facilities of the Indian health care system.

## Methods

For purposes of comparison, we considered all state pairs served by the Indian health care system that had notably differing HCV treatment eligibility in state Medicaid programs, were contiguous, and had similar Affordable Care Act Medicaid Expansion policies. For meaningful estimates, the states had to have at least 50,000 tribal registrants served.

Medicaid eligibility for HCV differ by criteria such as liver disease progression, substance use/sobriety requirements, and prescriber restrictions. We used an external, publicly available resource to assign a rating to HCV policy by state [5]. Tribal registrants are defined as enrolled members of the tribe within the geographic area served by the federal or tribal health facility. Registrant data was taken from the fiscal year 2018 IHS User Population Memorandum.

Prescription data were compiled via the IHS National Service Supply Center (NSSC), a central purchasing option for federal and tribal service units. These data record orders for HCV DAA prescriptions in 28-day units.

## Results

Contiguous state pairs that met the inclusion criteria were New Mexico/Arizona and Washington/Oregon. These dyads showed a significant difference in prescription rates per 100,000 inhabitants. States with a better Medicaid rating (WA, NM) had a prescription rate 4.7 times higher than their comparison states (Table 1).

**Table 1:** Direct acting antiviral (DAA) HCV prescriptions by population served, Indian Health Service and tribal health facilities, Washington and Oregon, New Mexico and Arizona, 2018

	Active Indian registrants	DAA prescriptions (28-day units)	Prescriptions per 100,000 population	Medicaid HCV drug access grade^a^	DAA prescriptions rate ratio (95% CI)
WA	127,167	116	91.2	A-	4.7 (2.6–8.5)
OR	61,530	12	19.5	D	
NM	366,163	114	31.1	A	4.7 (3.3–6.8)
AZ	601,817	40	6.7	C+	

^a^Stateofhepc.org

## Conclusions

Prescriptions per patient population were significantly higher in states with less restrictive Medicaid policies, with a rate ratio of nearly five times compared to neighboring states with more restrictive criteria. In the long term, policy-related treatment disparities may result in measurable differences by state in rates of hepatocellular carcinoma, liver transplants, and other adverse outcomes. The disparity in prescription rates between the two less restrictive states (OR and NM) may be attributable to differences in HCV burden, as IHS data document the Southwest as the region with the lowest apparent HCV seroprevalence [8].

These results have limitations, in that some prescriptions may not be documented by NSSC data, and that prescriptions ordered are a proxy for HCV treatment initiation. However, there is no known systemic difference in access to care, patterns of Medicaid usage, clinical capacity, or other variable between the facilities in each state dyad. Medicaid policy is the single greatest variable known to influence initiation of HCV treatment for the facilities in this review.

There are indications that some states are reducing HCV treatment restrictions. For example, Arizona recently eliminated fibrosis requirements, and Oregon eliminated fibrosis and sobriety barriers; these reforms may increase future prescription rates. However, a majority of state Medicaid programs retain varying degrees of eligibility criteria unique to HCV.

## Data Availability

Prescription data from Indian Health Service are generally not available to the public unless approved by Institutional Review Board.

## Abbreviations

AI/AN: American Indian/Alaska Native
AZ: Arizona
DAA: Direct acting antiviral
HCV: Hepatitis C virus
IHS: Indian Health Service
NM: New Mexico
NSSC: National Service Supply Center
OR: Oregon
WA: Washington

## References

[1] Centers for Disease Control and Prevention, Hepatitis C factsheet https://www.cdc.gov/hepatitis/hcv/hcvfaq.htm#section1, Accessed 1 Oct 2019.

[2] Ly KN, Hughes EM, Jiles RB, Holmberg SD (2016). Rising mortality associated with hepatitis C virus in the United States, 2003–2013. Clin Infect Dis.

[3] World Health Organization, Hepatitis C fact sheet, https://www.who.int/news-room/fact-sheets/detail/hepatitis-c, Accessed 1 Oct 2019.

[4] van der Meer AJ, Veldt BJ, Feld JJ, Wedemeyer H, Dufour JF, Lammert F (2012). Association between sustained virological response and all-cause mortality among patients with chronic hepatitis C and advanced hepatic fibrosis. JAMA.

[5] Backus LI, Belperio PS, Shahoumian TA, Mole LA (2018). Direct-acting antiviral sustained virologic response: impact on mortality in patients without advanced liver disease. Hepatology.

[6] Ward John W., Mermin Jonathan H. (2015). Simple, Effective, but Out of Reach? Public Health Implications of HCV Drugs. New England Journal of Medicine.

[7] Centers for Disease Control and Prevention, Surveillance for Viral Hepatitis, US, 2017 https://www.cdc.gov/hepatitis/statistics/2017surveillance/index.htm, Accessed 1 Oct 2019.

[8] Reilley Brigg, Leston Jessica, Doshani Mona, Haberling Dana L., Person Marissa, Weiser Thomas, Collier Melissa, Iralu Jonathan, Mera Jorge, Haverkate Rick (2018). Assessing Disparities in the Rates of HCV Diagnoses Within American Indian or Alaska Native Populations Served by the U.S. Indian Health Service, 2005–2015. Journal of Community Health.

[9] National Viral Hepatitis Roundtable, Center for Health Law and Policy Innovation, Harvard University, State of Hepatitis Medicaid Access,, stateofhepc.org, Accessed 1 July 2019.

[10] American Association for the Study of Liver Diseases and the Infectious Disease Society of America, When and in Whom to Initiate HCV Treatment, https://www.hcvguidelines.org/evaluate/when-whom, Accessed 2 Oct 2019.

